# Rapid Weight Loss, Central Obesity Improvement and Blood Glucose Reduction Are Associated with a Stronger Adaptive Immune Response Following COVID-19 mRNA Vaccine

**DOI:** 10.3390/vaccines10010079

**Published:** 2022-01-05

**Authors:** Mikiko Watanabe, Angela Balena, Davide Masi, Rossella Tozzi, Renata Risi, Alessandra Caputi, Rebecca Rossetti, Maria Elena Spoltore, Filippo Biagi, Emanuela Anastasi, Antonio Angeloni, Stefania Mariani, Carla Lubrano, Dario Tuccinardi, Lucio Gnessi

**Affiliations:** 1 Section of Medical Pathophysiology, Food Science and Endocrinology, Department of Experimental Medicine, Sapienza University of Rome, 00161 Rome, Italy; angela.balena@uniroma1.it (A.B.); davide.masi@uniroma1.it (D.M.); renata.risi@uniroma1.it (R.R.); alessandra.caputi@uniroma1.it (A.C.); Rebecca.rossetti@uniroma1.it (R.R.); mariaelena.spoltore@uniroma1.it (M.E.S.); biagi.1778697@studenti.uniroma1.it (F.B.); s.mariani@uniroma1.it (S.M.); Carla.lubrano@uniroma1.it (C.L.); lucio.gnessi@uniroma1.it (L.G.); 2 Department of Molecular Medicine, Sapienza University of Rome, 00161 Rome, Italy; rossella.tozzi@uniroma1.it; 3 Department of Experimental Medicine, Sapienza University of Rome, 00161 Rome, Italy; emanuela.anastasi@uniroma1.it (E.A.); Antonio.angeloni@uniroma1.it (A.A.); 4 Department of Endocrinology and Diabetes, University Campus Bio-Medico of Rome, 00128 Rome, Italy; d.tuccinardi@unicampus.it

**Keywords:** BMI, SARS CoV-2, infection, immunogenicity, diabetes, waist circumference, vaccination

## Abstract

Obesity is associated with a poor COVID-19 prognosis, and it seems associated with reduced humoral response to vaccination. Public health campaigns have advocated for weight loss in subjects with obesity, hoping to eliminate this risk. However, no evidence proves that weight loss leads to a better prognosis or a stronger immune response to vaccination. We aimed to investigate the impact of rapid weight loss on the adaptive immune response in subjects with morbid obesity. Twenty-one patients followed a hypocaloric, very-low-carbohydrate diet one week before to one week after the two mRNA vaccine doses. The diet’s safety and efficacy were assessed, and the adaptive humoral (anti-SARS CoV-2 S antibodies, Abs) and cell-mediated responses (IFNγ secretion on stimulation with two different SARS CoV-2 peptide mixes, IFNγ-1 and IFNγ-2) were evaluated. The patients lost ~10% of their body weight with metabolic improvement. A high baseline BMI correlated with a poor immune response (R −0.558, *p* = 0.013 for IFNγ-1; R −0.581, *p* = 0.009 for IFNγ-2; R −0.512, *p* = 0.018 for Abs). Furthermore, there was a correlation between weight loss and higher IFNγ-2 (R 0.471, *p* = 0.042), and between blood glucose reduction and higher IFNγ-1 (R 0.534, *p* = 0.019), maintained after weight loss and waist circumference reduction adjustment. Urate reduction correlated with higher Abs (R 0.552, *p* = 0.033). In conclusion, obesity is associated with a reduced adaptive response to a COVID-19 mRNA vaccine, and weight loss and metabolic improvement may reverse the effect.

## 1. Introduction

Early in the coronavirus disease 2019 (COVID-19) pandemic, obesity was suggested as a risk factor for the development of complications following infection [1,2], and substantial evidence has confirmed the preliminary observations [3], with visceral fat accumulation, chronic low-grade inflammation and metabolic derangement being key [4,5]. This is unsurprising as obesity is associated with immune dysfunction: those with obesity are more susceptible to infections and more at risk of developing complications on infection [6,7]. Interestingly, evidence also supports a link between obesity and poor seroconversion upon administration of some vaccines such as tetanus, hepatitis B and influenza virus [8,9], together with an increased risk of infection even when the seroconversion seems robust [10]. As we have previously reported, central obesity is also associated with lower antibody levels following an mRNA COVID-19 vaccine [11]. Nonetheless, Polack et al. reported that those with obesity seemed equally protected against the disease following the COVID-19 mRNA vaccine, although the data were only short term and caution was advised while interpreting them [12].

Given these preliminary findings, many public health campaigns and scientific societies have strongly advocated for weight loss in subjects with obesity, hoping that it will eliminate the reported risk [13]. However, to date, there is no evidence that weight loss is associated with either a better prognosis concerning COVID-19 or a more robust immune response to the vaccination. The metabolic derangements, low-grade inflammation and visceral fat accumulation typical of obesity, detrimental for the COVID-19 prognosis and possibly hindering seroconversion following the vaccine [11] will usually be improved on losing weight. However, weight loss is also recognized to blunt the immune system itself, especially when obtained through extreme caloric restriction, and it was previously reported that even moderate weight loss is associated with reduction of white blood cells’ number and function [14,15]. Altogether, the impact of weight loss on the immune system is multifaceted and unclear, and studies are needed to investigate its impact on the immune response following vaccination. Determining whether weight loss can boost the immune response to the COVID-19 vaccine is crucial, as should it not be the case, or worse, should weight loss be detrimental if obtained while undergoing the vaccine, current campaigns and scientific society recommendations should be taking this into strong consideration.

We, therefore, aimed to investigate the impact of rapid weight loss obtained through a hypocaloric, very-low-carbohydrate diet on the humoral and cell-mediated immune responses to a COVID-19 mRNA vaccine in subjects with morbid obesity.

## 2. Materials and Methods

### 2.1. Study Design

This was an observational prospective study investigating the impact on the adaptive immune response of rapid weight loss obtained during the two-dose mRNA COVID-19 immunization. The study lasted for five to seven weeks depending on the interval between the two vaccine doses, which were three to five weeks apart as per national policy changes. Patients were evaluated one week before the first inoculation, and a dietary intervention was started on enrolment. The patients were then followed up every two weeks, and one week after the second vaccine dose the patients came in for a final re-evaluation.

### 2.2. Study Population

Subjects were enrolled among those accessing the Center of High Specialization for the Cure of Obesity (CASCO), Polyclinic Umberto I, Rome, undergoing the COVID-19 vaccine in May–August 2021. The inclusion criteria were as follows: age ≥ 18 years old, stable body weight (less than 5 kg self-reported change during the preceding three months), willingness to undergo vaccination, body mass index (BMI) ≥ 35 kg/m^2^ with at least one obesity-related complication or BMI ≥ 40 kg/m^2^ alone. The exclusion criteria were: immunodepression, use of medications known to impact the immune system, use of anti-obesity medications or others potentially affecting body weight or SGLT2 inhibitors, pregnancy, lactation, previous SARS-CoV-2 infection, glomerular filtration rate (GFR) < 60 mL/min, insulin-dependent diabetes or history of hyperuricemia. Data about demographic characteristics were collected through a structured interview. The study was approved by the local IRB (prot. CE6228) and conducted in accordance with the Declaration of Helsinki and Good Clinical Practice. Written informed consent was obtained from all study participants before enrollment.

### 2.3. Dietary Intervention

The energy requirement was calculated by adjusting for the physical activity level [16]. Patients were prescribed a calorie deficit of ~400 kcal (prescribed intake 1000–1700 kcal for all) with a carbohydrate intake of <50 g/day. The protein intake was 1.2–1.4 g/kg ideal body weight, and fat made up for the rest of the caloric intake. Participants were counselled to drink 2 L of water/day and choose vegetarian and healthy sources of fat; protein was to mainly come from fish, eggs, fresh dairy products and lean meat. Patients were counselled by a trained dietician every two weeks, and compliance was assessed through a three-day dietary recall task at each visit and beta-hydroxybutyrate (BHB) and acetoacetate (AA) assessments. 

### 2.4. Vaccination Procedure and Blood Collection

All patients received two COVID-19 vaccine inoculations, separated by 21–35 days (BNT162b2, Pfizer-BioNTech). Before the first, all patients underwent a blood draw, and a second one was collected one week after the second inoculation. The samples were then centrifuged and the serum/plasma was kept at −80 °C until further analysis.

### 2.5. Biochemical Measures

Routine biochemical tests were handled according to standard operating procedures, from fasting (12 h) blood samples (electrolytes, glucose, insulin, lipid profile (triglycerides and total, high-density lipoprotein (HDL) and low-density lipoprotein (LDL) cholesterol), creatinine, blood urea nitrogen (BUN), alanine transferase (ALT), aspartate transaminase (AST), uric acid and estimated GFR). 

The capillary BHB was measured using a commercially available device (VTRUST TD4279, Biochemical Systems International S.p.A., Milan, Italy). The urine semiquantitative AA was assessed through reactive strips (One step K, DFI Co., Ltd, Gimhae-si, Gyeongsangnam-do, Korea). 

Anti-SARS-CoV2 antibodies were measured through a commercially available assay (Elecsys^®^ anti-SARS-CoV-2 assay, Roche Diagnostics, Switzerland), detecting antibodies against the SARS-CoV-2 spike (S) antigen (Ag) in a sandwich electrochemiluminescence assay (ECLIA) [17]. The total antibodies against the SARS-CoV2 nuclear (N) protein were assessed to exclude undiagnosed natural infection. 

The T-cell reactivity to SARS-CoV-2 was assessed through a commercially available interferon-γ (IFN-γ) release assay (QuantiFERON^®^ SARS-CoV-2 RUO, Qiagen, Hilden, Germany). Briefly, heparinized whole blood was incubated at 37 °C for 24 h within 8 h of its collection in different tubes: a Mitogen tube containing non-specific T-cell stimulating antigens (positive control); a Nil tube with no antigen added (negative control); Ag1 and Ag2 containing two different antigen peptide pools specific to SARS-CoV-2 to stimulate lymphocytes. Following incubation, the samples were centrifuged to harvest the stimulated plasma. IFN-γ was quantified using a QuantiFERON^®^ IFN-γ ELISA assay (Qiagen, Hilden, Germany). The IFN-γ levels obtained from the Mitogen, Ag1 and Ag2 tubes were corrected for the background (negative control tubes) and denominated PC, IFNγ-1 and IFNγ-2. In line with the tuberculosis test recommendations, Nil-values of <0.7 IU/mL and Nil subtracted PC values of ≥0.5 IU/mL were considered as the reference standards.

### 2.6. Anthropometric and Body Composition Assessment

The body weight was measured using a balance-beam scale (Seca GmbH & Co., Hamburg, Germany). The height was rounded to the closest 0.5 cm. The waist circumference was measured midway between the lower rib and the iliac crest, and the hip circumference at the level of the widest circumference over the great trochanters to the closest 1.0 cm. The systolic and diastolic blood pressure (BP) were measured using an automated digital device. As this dietary intervention is known to reduce the BP, those on antihypertensives were advised to contact the study team in case of BP reduction. Medications were adjusted accordingly. 

### 2.7. Adverse Events

Adverse events to the vaccine and the dietary intervention were recorded through a structured physician-administered questionnaire. The timing, duration, type and entity of adverse events were recorded at each follow-up visit.

### 2.8. Statistics

The Statistical Package for Social Sciences (SPSS), v.20 was used. Results are presented as the median and interquartile range. As the variables were not normally distributed, non-parametric analysis was conducted. A Spearman correlation method was used to analyze the correlation between continuous variables. Partial correlation analysis was used to adjust for covariates. To build a multivariate linear regression model with IFNγ-2 as the dependent variable and the baseline BMI, weight loss and age as regressors, an enter method approach was used. Expecting a strong correlation between the BMI and humoral response based on the current literature, a sample size of 19 was calculated, with an alpha of 0.05 and beta of 0.2. Accounting for a 20% dropout rate, 24 patients were enrolled. The results were considered statistically significant when *p* < 0.05.

## 3. Results

### 3.1. Study Population

Twenty-four subjects were enrolled. Of these, two dropped out due to personal reasons and one due to the occurrence of adverse events following the first vaccination, posing a contraindication to the second dose. The baseline characteristics are summarized in Table 1. Briefly, the median age was 51 years, 71.4% were female, the median BMI was 40.95 kg/m^2^ and all were Caucasian. All patients had central obesity, with an average waist and hip circumference of 122.5 cm and 135 cm, respectively. Eleven patients suffered from hypertension, two had diabetes, 13 were dyslipidemic, five were present smokers and eight were ex-smokers (Table 1). 

### 3.2. Dietary Intervention Metabolic Efficacy

After the dietary intervention, all patients were reassessed, and the anthropometric parameters are shown in Table 1. The patients lost ~11.5 kg. The excess weight loss was ~30%, with a weight reduction of ~10%. The systolic BP significantly improved, whereas the diastolic BP was unchanged, and seven out of twelve patients (58%) reduced or stopped antihypertensives. Glucose and insulin decreased, as d total cholesterol and triglycerides (Table 1). The median BHB and AA were 0.78 mmoL/L and 0.67 mmoL/L, respectively, reflecting excellent dietary adherence (Table 1).

### 3.3. Dietary Intervention Safety

No major adverse events were reported. Mild adverse events were reported at some point of the intervention: 13% reported occasional upper gastrointestinal symptoms (nausea, vomiting, gastric reflux, heartburn), 21% occasional lower gastrointestinal symptoms (bloating, constipation, diarrhea, abdominal pain) and 29% occasional fatigue or muscle weakness or palpitations. An increase in fiber or water intake, change in food quality or frequency or adjustment of antihypertensive therapy effectively resolved the symptoms. The electrolytes remained unchanged, as did the BUN, liver function and uric acid. The creatinine increased significantly, but all patients remained in the normal reference range. A reduction in HDL cholesterol was observed (Table 1).

### 3.4. Vaccine Adverse Events

Following the first COVID-19 vaccine dose, nine patients reported mild events (pain at the site of injection, myalgia, fatigue or low fever (<38 °C)). Following the second dose, eleven patients reported mild events. No correlation was observed between the occurrence of adverse events following the first or second dose and the humoral or cell-mediated immune response (data not shown).

### 3.5. Adaptive Immune Response

The immune response to vaccination was assessed one week after the second vaccine inoculation. No patient was anergic for either the humoral or cell-mediated response, as reflected by the presence of seroconversion (Abs titer > 0.823 BAU/mL) and a Nil subtracted PC well above 0.5 IU/mL for all subjects. The median antibody titer (Abs) was 2070 BAU/mL, while the median IFNγ-1 and IFNγ-2 concentrations were 0.86 IU/mL and 1.82 IU/mL, respectively. 

**Figure 1:**
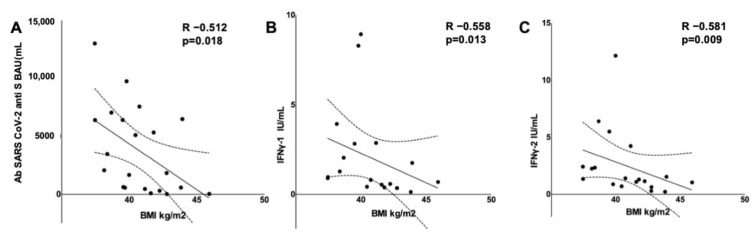
Scatterplots showing that baseline BMI is inversely correlated with humoral (**A**) and cell-mediated immune response after an mRNA COVID-19 vaccination (**B**,**C**).

The baseline BMI was inversely correlated with the cell-mediated and humoral responses (R −0.558, *p* = 0.013 for IFNγ-1; R −0.581, *p* = 0.009 for IFNγ-2; R −0.512, *p* = 0.018 for Abs; Figure 1A–C). Age was inversely correlated with IFNγ-2 (R −0.513, *p* = 0.025), but not with the IFNγ-1 or Abs titer concentration (R −0.32, *p* = 0.182; R −0.123, *p* = 0.595). The time from the first dose was not associated with a differential immune response. No difference was observed in the humoral or cell-mediated response between males and females, over and below the age median, hypertensive and non-hypertensive, smoker and non-smoker and dyslipidemic and non-dyslipidemic (data not shown). 

**Figure 2:**
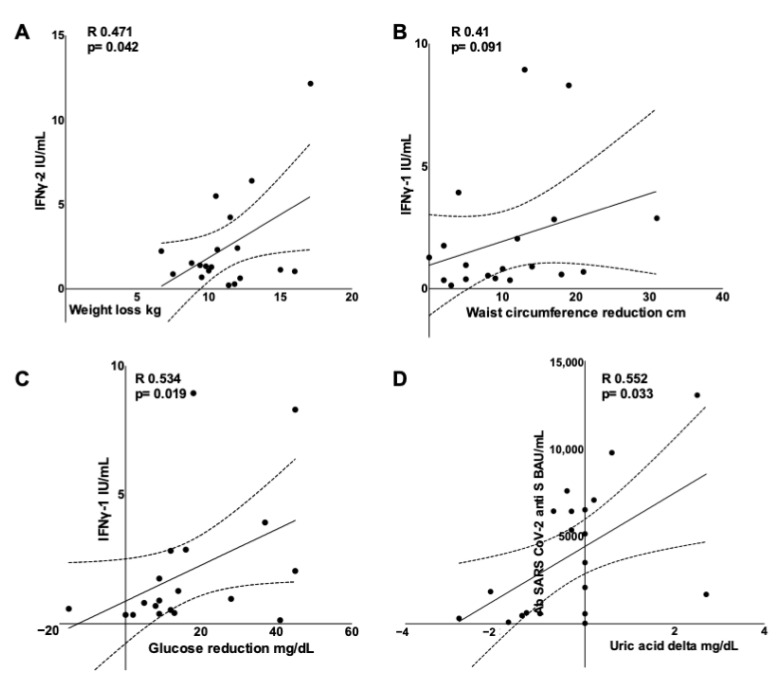
Scatterplots showing that metabolic improvement on dieting is directly correlated with the adaptive immune response after an mRNA COVID-19 vaccination. IFNγ-1 loss is directly correlated with (**A**) weight loss, (**B**) waist circumference reduction (trend) and (**C**) glucose reduction, while anti SARS CoV-2 antibodies are directly correlated with uric acid change (**D**).

Weight loss showed a direct correlation with the IFNγ-2 concentration (R 0.471, *p* = 0.042, Figure 2A) but not with IFNγ-1 or Abs (R 0.031, *p* = 0.900 and R −0.238, *p* = 0.302, respectively). A multivariate analysis including IFNγ-2 as the dependent variable and the baseline BMI, weight loss and age as regressors showed that both the baseline BMI and weight loss were significant predictors (B = −0.668, *p* = 0.013 and B = −0.635 *p* = 0.005, respectively), whereas age was not (B = −0.069, *p* = 0.198; R^2^ 0.562, *p* = 0.005). Similarly, central obesity improvement as reflected by waist circumference reduction was directly, although not significantly, correlated with the IFNγ-1 concentration (R 0.41, *p* = 0.091, Figure 2B) but not with IFNγ-2 or Abs (R 0.304, *p* = 0.221 and R 0.108, *p* = 0.649, respectively).

Blood glucose reduction was directly correlated with the IFNγ-1 concentration (R 0.534, *p* = 0.019, Figure 2C) but not with the IFNγ-2 concentration or Abs (R 0.282, *p* = 0.242 and R 0.237, *p* = 0.301, respectively). A partial correlation analysis showed that the direct correlation between IFNγ-1 and glucose reduction remained significant after adjusting for weight loss and waist circumference reduction (R 0.483, *p* = 0.042 and R 0.497, *p* = 0.042, respectively). Uric acid reduction significantly correlated with Abs titers (R 0.552, *p* = 0.033, Figure 2D).

## 4. Discussion

We report that a higher baseline BMI is associated with reduced humoral and cell-mediated responses to a COVID-19 mRNA vaccine, while weight loss and blood glucose and urate reduction seem to reverse this negative effect in subjects with obesity.

Obesity is characterized by a reduced number and function of B-cells [18]. Moreover, depletion of CD8 memory T lymphocytes is observed, together with a decreased number and function of CD4+ T lymphocytes and increased number of regulatory T cells (Treg), possibly caused by leptin [19,20] and insulin resistance [21,22]. As obesity is associated with shorter telomeres due to oxidative stress and chronic low-grade inflammation [23,24], the shorter telomere length of T cells might hinder effector memory T cells’ (T_EM_) proliferation and development on infection and vaccination [25]. Excess weight also enhances the expression of programmed cell death protein 1 and programmed death-ligand 1 in T_EM_ cells. This, in turn, diminishes T_EM_-cell responsiveness [26], potentially hindering long-term protection. 

Regarding the efficacy of vaccines in the obese population, evidence suggests possible poor seroconversion [8], and even when seroconversion is robust, obesity may lead to an increased risk of infection [10], possibly due to reduced T_EM_-cell activity long-term [27]. Moreover, the thicker arm fat pad of patients with obesity may lead to reduced immunogenicity when the vaccine is deposited in the fat rather than in the subject’s muscle, which could be avoided by using longer needles in certain individuals [28,29]. Our finding was expected and in line with previous reports of lower antibody levels following COVID-19 vaccination in those with excess weight [11,30,31]. Our results also showed that the cell-mediated response, in addition to the humoral response, is reduced as well. However, it should be highlighted that the immunogenicity of the mRNA COVID-19 vaccines is exceptionally high, as confirmed by the net reduction of infection and hospitalization following inoculation [32,33], and a lower adaptive response may be sufficiently effective, at least in the short term.

In our study, a hypocaloric very-low-carbohydrate approach initiated one week before the first dose and continued until one week after the second dose was effective in inducing both rapid metabolic improvement and weight loss in subjects with morbid obesity undergoing a COVID-19 mRNA vaccination. Very-low-carbohydrate diets are often used to obtain rapid and significant weight loss in their very-low-calorie form [34] with an excellent safety profile [35]. Nonetheless, when extremely hypocaloric, they may reduce chemotaxis and microbial killing [36]. More generally, severe acute malnutrition is associated with a blunted immune response [37], whereas mild energy restriction has been shown to improve the immune response to viral agents in mouse models [38]. Hence, we opted for a relatively mild hypocaloric dietary regimen. 

Concerning metabolic efficacy, profound weight loss together with lipid and glucose metabolism improvements were observed, similar to the effects of very-low-calorie diets leveraging meal replacements [39,40,41,42]. This diet was a cheap, effective and well-tolerated way to obtain rapid weight loss and metabolic improvement. A small yet significant creatinine increase was observed, likely due to the increased protein intake. The GFR was in the normal range for all participants throughout the study, but its long-term significance should be clarified. It is safe to assume that such a dietary intervention is safe short-term in patients with no chronic kidney disease, but caution is otherwise warranted. HDL cholesterol was significantly decreased following the dietary intervention, likely due to the absolute reduction in fat intake and lack of increase in physical activity levels. This is in line with previous findings, and its long-term significance remains unknown. Short term, the HDL decrease may not represent a significant clinical risk, but further studies are needed. 

The weight loss obtained in this study was directly associated with a more robust cell-mediated adaptive response, and a trend was seen towards a correlation with central obesity improvement. The impact of weight loss on immune function is not well-established. It was previously reported that the activity of peripheral blood mononuclear cells (PBMCs) in morbid obesity is blunted, as reflected by reduced IFNγ and the monocyte chemotactic protein-1 (MCP-1), but weight loss is capable of restoring the ability of stimulated PBMCs to produce MCP-1 and IFNγ [43]. Similarly, patients undergoing significant weight loss thanks to bariatric surgery have an increased activity of natural killer (NK) [44]. Going in the opposite direction, a hypocaloric regimen coupled with orlistat caused a reduction in NK cells in women with obesity, but not other immune cells compared to the control on an *ad libitum* diet [45]. Proinflammatory markers’ and lymphocytes’ reduction was finally interpreted as an improvement following moderate weight loss in patients with obesity and glucose metabolism impairment [46]. Furthermore, patients with visceral obesity have elevated tumor necrosis factor alpha (TNFα) levels compared to those with subcutaneous obesity, suggesting that the visceral depot contributes significantly to circulating TNFα levels [47]. However, when following a very-low-calorie diet, patients with obesity had reduced TNFα levels even though not to the average level exhibited by lean controls [48].

No evidence is available regarding the immunogenic impact of weight loss obtained in the period around a vaccination procedure. It seems that a moderate calorie restriction might have been beneficial, confirming studies investigating its impact on the immune system. Moreover, beyond the metabolic improvement, very-low-carbohydrate diets have shown to be immunomodulatory in mouse models, as these were found to be capable of enhancing tumor-reactive CD8+ T cell and NK cell activity in glioblastoma [49] and were protective against influenza virus via an increased number and activity of protective γδ T cells in the lungs [50], a finding that was confirmed on infection with the murine equivalent of SARS-CoV-2 [51]. Moreover, isocaloric ketogenic diets have been proven effective in improving the COVID-19 prognosis in human subjects via immune modulation [52,53].

Glucose reduction was strongly associated with a better cell-mediated response to vaccination, independent of weight loss or central obesity improvement. No studies are available regarding the impact of glucose metabolism improvement on vaccination, but excess glucose is known to be detrimental to the immune system [54]: a reduction in the number and function of NK and Treg cells [55,56] and deficient T activation were observed [57,58]. Rapid glucose normalization, as seen in our population and as commonly reported on a very-low-carbohydrate diet, might have boosted the adaptive response even before weight loss.

Finally, urate reduction was associated with a higher humoral response to the vaccination. Although no evidence is available on its impact on vaccines’ immunogenicity, some studies suggest that hyperuricemia is associated with a dysfunctional and at times blunted immune function. Most report a bidirectional link with chronic low-grade inflammation [59], but it was also reported that patients with hyperuricemia exhibit a lower number of NK cells with impaired function [60], and a hyperuricemia mouse model showed a reduced number and function of tumor-specific CD8+ [61], suggesting that lower urate may aid an appropriate immune response.

Our study has several limitations. First, the interval between the first and second dose varied between three and five weeks as per national policy change over time, introducing time as a possible bias. However, no difference was observed between those undergoing the three- or five-week protocol with respect to immunogenicity. Second, the sample size of our study population was small, and occasional values were missing completely at random, leading to some pairwise exclusions in the statistical analysis [62]. However, in all the analyses conducted other than the correlation between uric acid reduction and IFNγ (*n* = 18), a sample size of at least 19 was maintained, as per the *a priori* sample size calculation. Further to this, some outliers were present, which were re-analyzed to confirm that they were not imputable to inaccurate collection, analysis or reporting of data. The identified outliers were maintained as they may capture valuable information, such as the presence of super responders to the vaccination. However, larger studies are needed to confirm our findings. Of note, the patients were highly homogeneous in terms of demographic and anthropometric characteristics, and dietary compliance was ensured throughout the study, limiting the heterogeneity of the results. Third, the study endpoint was only one week after the second dose, and the long-term immune response was, therefore, not investigated. The study endpoint was picked to evaluate the acute weight loss effect, foreseeing a loss of dietary compliance over time.

Our study also features some strengths. This is the first study investigating the immunogenic impact of weight loss obtained during vaccine administration to the best of our knowledge. Moreover, not only was the humoral response assessed but so were cell-mediated outcomes. In addition, patients with morbid obesity were enrolled, a subpopulation often underrepresented in clinical trials and most at risk of developing infection-related complications. Also, a cheap and effective dietary intervention not leveraging costly meal replacements was adopted, making the intervention feasible for most individuals. 

## 5. Conclusions

In conclusion, our results suggest that morbid obesity may hinder the immune response to COVID-19 mRNA vaccines, although the reduced response may still be protective against infection or at least its complications. Rapid weight loss, with its metabolic improvement, seems effective at enhancing the adaptive response and may be considered, especially in the case of morbid obesity. Further studies evaluating the long-term immune response to mRNA vaccines and other vaccine types are needed, and a controlled trial evaluating whether the macronutrient ratio, rather than simple weight loss, played a role in the immunogenicity of the intervention is warranted.

## Figures and Tables

**Table 1:** Study population demographic, anthropometric and biochemical data.

	Pre			Post			*p*
*n*		21			21		
Female, *n* (%)	15(71.4)						
Age (years)	51.50	41.50	55.25				
Weight (kg)	111.00	106.25	117.88	100.15	94.33	106.30	0.001
BMI (Kg/m^2^)	40.95	39.30	42.76	36.83	34.93	38.23	0.001
Waist circumference (cm)	122.50	117.75	136.25	114.00	106.00	119.25	0.001
Hip circumference (cm)	132.00	125.75	134.75	121.00	115.50	128.00	0.001
Waist-to-hip ratio	0.96	0.85	1.04	0.96	0.83	1.03	0.093
Systolic BP (mmHg)	130.00	120.00	140.00	120.00	110.00	130.00	0.024
Diastolic BP (mmHg)	80.00	70.00	91.25	75.00	70.00	80.00	0.325
Heart rate (bpm)	77.50	70.75	85.00	70.00	63.50	78.50	0.345
Glucose (mg/dL)	99	97	117	86	85	96.5	0.000
Insulin (µU/mL)	24.2	16.35	35.05	11.5	9.275	15.775	0.000
BUN (mg/dL)	31.8	25.05	37.95	33.6	27	37.5	0.191
Creatinine (mg/dL)	0.79	0.695	0.8975	0.82	0.7675	0.905	0.013
Sodium (mmoL/L)	140	137.75	141.25	141	137.75	143	0.274
Potassium (mmoL/L)	4.35	3.875	4.5	4.3	4.125	4.475	0.809
AST (U/L)	19	16.75	21	20	16	23.75	0.822
ALT (U/L)	24	18	28.25	21	17.25	25	0.156
Total cholesterol (mg/dL)	197	173.75	221.75	179	152.75	207.5	0.000
LDL cholesterol (mg/dL)	115	99.5	141	113	88.75	151	0.360
HDL cholesterol (mg/dL)	49	39.25	59.5	43	34	50.75	0.001
Triglycerides (mg/dL)	116	77	179.75	97	68	113.5	0.002
Uric acid (mg/dL)	5.5	5.275	6.25	6.7	5.85	6.8	0.177
Capillary BHB (mmoL/L)				0.78	0.63	0.97	
U. acetoacetate (mmoL/L)				0.67	0.46	2.42	
IFNγ-1 (IU/mL)				0.86	0.41	2.84	
IFNγ-2 (IU/mL)				1.32	0.83	2.87	
Anti S Abs (BAU/mL)				2070	519	6464	
Diabetes, *n* (%)	2(9.5)						
Hypertension, *n* (%)	11(52.4)						
Dyslipidemia, *n* (%)	13(61.9)						
Smoking habit, *n* (%)	5(23.8)						

Data are expressed as median and 25th–75th percentile, *p* is from a Wilcoxon signed-rank test. BP, blood pressure; BUN, blood urea nitrogen; AST, aspartate aminotransferase; ALT, alanine aminotransferase; LDL, low-density lipoprotein; HDL, high-density lipoprotein; BHB, beta-hydroxybutyrate; U., urinary; IFNγ-1, interferon γ peptide mix 1; IFNγ-2, interferon γ peptide mix 2; anti S Abs, anti-SARS-CoV-2 spike protein antibodies.

## Data Availability

Data will be made available on reasonable request to the corresponding author.
